# Complete genome sequence of *Sphingobacterium kitahiroshimense* strain Ss8-5, isolated from a sclerotium of *Sclerotinia sclerotiorum*

**DOI:** 10.1128/mra.00846-26

**Published:** 2026-08-11

**Authors:** Nozomu Iwabuchi

**Affiliations:** 1 Graduate School of Environment and Information Sciences, Yokohama National University 13154, Yokohama, Japan; USDA-ARS San Joaquin Valley Agricultural Sciences Center, Parlier, California, USA

**Keywords:** *Sclerotinia sclerotiorum*, sclerotia, *Sphingobacterium*, complete genome, PacBio

## Abstract

I report the complete genome sequence of *Sphingobacterium kitahiroshimense* strain Ss8-5, isolated from a sclerotium on a cabbage head affected by *Sclerotinia* rot. The complete genome consists of a single circular chromosome of 6.1 Mb with a GC content of 36.3%.

## ANNOUNCEMENT

Fungal sclerotia of the plant-pathogenic fungus *Sclerotinia sclerotiorum* serve as critical long-term survival structures in soil (1), and their interacting bacterial communities can significantly influence fungal viability and ecological roles (2). To investigate these micro-ecological interactions within fungal niches, a cabbage head (cultivar Kinkei no. 201) affected by *Sclerotinia* rot was collected in Kanagawa, Japan (35.373154°N, 139.470432°E), in April 2026. A bacterial strain, Ss8-5, was isolated from a sclerotium obtained from the collected cabbage head. The sclerotium was surface-sterilized with 70% ethanol for 1 min, 1% NaClO for 2 min, and rinsed three times with sterile water. Then, the sclerotium was homogenized in sterile water, and the resulting homogenate was plated on yeast extract peptone (YP) agar and incubated at 25°C for 2 days. Crude DNA was extracted by thermal lysis, and the 16S rRNA gene was amplified using universal primers 8F (5′-AGAGTTTGATCCTGGCTCAG-3′) and 1492R (5′-GGTTACCTTGTTACGACTT-3′), followed by Sanger sequencing (Eurofins Genomics, Japan). BLASTn analysis (3) based on the 16S rRNA gene sequence showed the highest similarity to *Sphingobacterium kitahiroshimense* (GenBank accession no. MK402063.1), a species originally isolated from soil (4).

For genomic DNA extraction, strain Ss8-5 was cultivated in YP broth at 28°C. Genomic DNA was isolated using the NucleoBond HMW DNA Kit (Macherey-Nagel, Germany) according to the manufacturer’s protocol. High-throughput sequencing and primary bioinformatic analyses were performed by Bioengineering Lab Co., Ltd. (Japan). DNA concentration and quality were assessed using a Quantus Fluorometer with the QuantiFluor dsDNA System (Promega, USA) and a 5200 Fragment Analyzer System with the Agilent HS Genomic DNA 50 kb Kit (Agilent Technologies, Inc., USA). DNA was sheared to approximately 10–25 kb using a Megaruptor 3 system (Diagenode, Belgium). A PCR-free library was constructed using the SMRTbell Prep Kit 3.0 (PacBio, USA). Polymerase-template complexes were bound using the Revio SPRQ Polymerase Kit (PacBio), and sequencing was performed on the PacBio Revio platform. PacBio sequencing generated 86,121 raw reads with a read N50 of 15,963 bp. Primary data processing was conducted using SMRT Link v25.2.0.266456 (PacBio) to remove overhang adapters, generate consensus sequences (CCSs), and retain reads with a mean quality score of ≥20 as HiFi reads. The HiFi reads were further filtered using Filtlong v0.2.1 (https://github.com/rrwick/Filtlong) to remove reads shorter than 1,000 bp and the lowest 10% of reads by quality. The processed HiFi reads were *de novo-*assembled using Flye ver. 2.9.3-b1797 (5). The assembly was circularized and rotated to begin with *dnaA* on the forward strand using Circlator v1.5.5 (6). Default parameters were used for all software.

The complete genome contains a single circular chromosome (6,100,171 bp) with 36.3% GC content and 203× coverage depth. CheckM2 analysis (v.1.0.1) (7) confirmed 100% completeness with 0.28% contamination. DFAST v1.6.0 (8) predicted 5,171 genes, including 5,057 protein-coding genes, 25 rRNAs, and 89 tRNAs. The average nucleotide identity (ANI) values between strain Ss8-5 and previously known *Sphingobacterium kitahiroshimense* strains ranged from 96.21% to 97.61%, calculated using the JSpeciesWS (9), supporting the taxonomic assignment based on the 16S rRNA gene sequence.

## Data Availability

The complete genome sequence has been deposited in the DNA Data Bank of Japan (DDBJ) under accession number AP050807. Raw sequence data have been deposited in the DDBJ/Sequence Read Archive under accession number DRR1070523.
